# Zoanthropy in confinement

**DOI:** 10.1192/j.eurpsy.2021.2099

**Published:** 2021-08-13

**Authors:** M.F. Tascon Guerra, M.V. López Rodrigo

**Affiliations:** Psychiatry, Hospital Ntra Sra del Prado, Talavera de la Reina, Toledo, Spain

**Keywords:** Zoanthropy, psychosis, Hear-impairment, confinement

## Abstract

**Introduction:**

Zoanthropy is a mental disorder in which a patient believes to be an animal. These patients believe they have morphed into another species and began to act like such. Several types of zoanthropy have been described. Mental disorders can be triggered by stressful life events in patients with certain vulnerability. Hearing impairment as a risk factor for psychosis has been suggested in Psychiatry research. The potential mechanisms underlying this association included loneliness, diminished theory of mind, disturbances of source monitoring and top-down processing and deafferentiation.

**Objectives:**

This case presents a patient, with no history of psychiatric diseases, who developed the delirious of being a dog during Codiv-19 quarantine.

**Methods:**

Previously healthy, sixty-year-old woman, with poor hearing, was taken to the Hospital for altered behavior after the confinement was stated. A low back pain started which derived on walking difficulties. The transformation begun and she started walking on four legs, barked and even pooped like a dog. Physical examination was normal. Mental exam revealed presence of delusion. Blood tests and brain imaging revealed no abnormalities. A treatment based on long-acting injectable aripiprazole was started.

**Results:**

Within 4 months of treatment, her mental state improved by attenuation of psychotic symptoms.

**Conclusions:**

Sensory impairment and social isolation, have been associated with late-onset psychosis, but appear to exert a nonspecific influence on vulnerability. Early assessment and treatment of hearing impairment in patients with high risk of psychosis may be essential in psychosis treatment and prevention.

**Disclosure:**

No significant relationships.

